# Immunogenicity and protective efficacy of a recombinant protein subunit vaccine and an inactivated vaccine against SARS-CoV-2 variants in non-human primates

**DOI:** 10.1038/s41392-022-00926-y

**Published:** 2022-03-03

**Authors:** Qian He, Qunying Mao, Xiaozhong Peng, Zhanlong He, Shuaiyao Lu, Jialu Zhang, Fan Gao, Lianlian Bian, Chaoqiang An, Wenhai Yu, Fengmei Yang, Yanan Zhou, Yun Yang, Yanyan Li, Yadi Yuan, Xujia Yan, Jinghuan Yang, Xing Wu, Weijin Huang, Changgui Li, Junzhi Wang, Zhenglun Liang, Miao Xu

**Affiliations:** 1 Division of Hepatitis and Enterovirus Vaccines, Institute of Biological Products, National Institutes for Food and Drug Control; NHC Key Laboratory of Research on Quality and Standardization of Biotech Products; NMPA Key Laboratory for Quality Research and Evaluation of Biological Products, Beijing, 102629 People’s Republic of China; 2 Institute of Medical Biology, Chinese Academy of Medicine Sciences & Peking Union Medical College, Kunming, 650118 People’s Republic of China

**Keywords:** Vaccines, Infection

## Abstract

Emerging SARS-CoV-2 variants and the gradually decreasing neutralizing antibodies over time post vaccination have led to an increase in incidents of breakthrough infection across the world. To investigate the potential protective effect of the recombinant protein subunit COVID-19 vaccine targeting receptor-binding domain (RBD) (PS-RBD) and whole inactivated virus particle vaccine (IV) against the variant strains, in this study, rhesus macaques were immunized with PS-RBD or IV vaccine, followed by a Beta variant (B.1.351) challenge. Although neutralizing activity against the Beta variant was reduced compared with that against the prototype, the decreased viral load in both upper and lower respiratory tracts, milder pathological changes, and downregulated inflammatory cytokine levels in lung tissues after challenge demonstrated that PS-RBD and IV still provided effective protection against the Beta variant in the macaque model. Furthermore, PS-RBD-induced macaque sera possessed general binding and neutralizing activity to Alpha, Beta, Delta, and Omicron variants in our study, though the neutralizing antibody (NAb) titers declined by varying degrees, demonstrating potential protection of PS-RBD against current circulating variants of concern (VOCs). Interestingly, although the IV vaccine-induced extremely low neutralizing antibody titers against the Beta variant, it still showed reduction for viral load and significantly alleviated pathological change. Other correlates of vaccine-induced protection (CoP) like antibody-dependent cellular cytotoxicity (ADCC) and immune memory were both confirmed to be existing in IV vaccinated group and possibly be involved in the protective mechanism.

## Introduction

As of September 2021, a total of six COVID-19 vaccine candidates have been placed on the Emergency Use List by the WHO for the worldwide vaccination project, since the efficacy of the vaccines submitted to WHO met the recommended efficacy standards.^1^ More than 6 billion vaccine doses have already been administered worldwide. However, weekly new confirmed cases (0.7 million) of COVID-19 in August 2021 did not show any reduction compared to that during the same time last year (0.2 million),^2,3^ when none of the current vaccine candidates had been approved by National Regulation Agencies. Real-world data have aggravated vaccine anxiety and hesitation.

A variety of newly emerging variants, combined with gradually decreasing neutralizing antibodies, have triggered a resurgence of the epidemic. Some variants, such as Alpha (B.1.1.7), Beta (B.1.351), Gamma (P.1), and Delta (B.1.617.2) are characterized by increased infectivity,^4–7^ pathogenicity,^8–10^ or immune escape,^11–14^ posing a great threat to global health, and are defined as variants of concern (VOCs) by the WHO.^15^ VOCs have caused various degrees of decline in both neutralizing ability and protective efficacy of vaccines. In particular, neutralizing antibody (NAb) titers induced by mRNA-1273, BNT162b2, and ChAdOx1 declined 3.5–3.8, 3.0–16.0, and 9.0–11.0 fold against B.1.351,^16–19^ and approximately 6.8, 1.4–8.4, and 2.5–9.0 fold against B.1.617.2, respectively.^20–23^ The vaccine efficacy (VE) of ChAdOx1 and BNT162b2 decreased to 10.4% and 75% against B.1.351, and 59.8% and 87.9% against B.1.167.2, respectively.^24,25^ All these data together indicated that the Beta variant maximally affected both NAb and VE of the approved vaccines.

The recombinant protein vaccine is currently the most researched COVID-19 vaccine platform, based on the WHO statistics.^26^ Recombinant protein vaccines, together with inactivated virus vaccines, are exogenous vaccines, whose targeted antigens are delivered into cells rather than being expressed in the cytoplasm. They mainly induce humoral immunity, owing to the lack of MHC-1 pathways. Protein subunit vaccine-like ZF2001 and SCB-2019, and inactivated vaccines have been administered at 1–3 doses in a large-scale population worldwide. Current data showed effective protection of protein subunit and inactivated vaccine against epidemic delta strain in humans, and an overall VE of 77.54%, 79.1%, and 59% for ZF2001, SCB-2019, and inactivated vaccines has been reported, respectively.^27,28^ Since newly emerging variants with higher immune escape ability might challenge the immune-protective barrier developed by such vaccines, extensive study and clarification of the potential protective effects of protein subunit vaccines and inactivated vaccines against beta variants are imperative. This may help to predict the protection offered by protein subunit vaccines and inactivated vaccines against other VOCs prevalent today or upcoming in the future. Considering the prolonged time, extensive labor, and heavy cost of clinical trials, along with the complex subject background and diagnostic criteria in different clinical trials, intervention experiments with non-human primates (NHPs) are appropriate for evaluating the potential protection efficacy in an urgent time. Furthermore, a better understanding of the interrelationships among vaccination, the immune response, protection, and clinical outcomes is of great significance for the development of surrogates and optimization of COVID-19 vaccines. The levels of antibody titers that could provide adequate protection against SARS-CoV-2 infection are still obscured. Challenging macaque with SARS-CoV-2 after immunization with various vaccines could be one rationale model to study correlates of vaccine-induced protection.

In this study, the rhesus macaque model was used to explore the cross-neutralizing ability of recombinant protein subunit COVID-19 vaccine targeting receptor-binding domain (PS-RBD) and IV vaccines against different VOCs. More importantly, a B.1.351 challenge model was established in rhesus macaques, and the protective effect of the aforementioned vaccines against B.1.351 was evaluated. Furthermore, the correlation between vaccine protection with NAb and other possible CoPs was analyzed.

## Results

### Immunogenicity of PS-RBD in young rhesus macaques

**Fig. 1:**
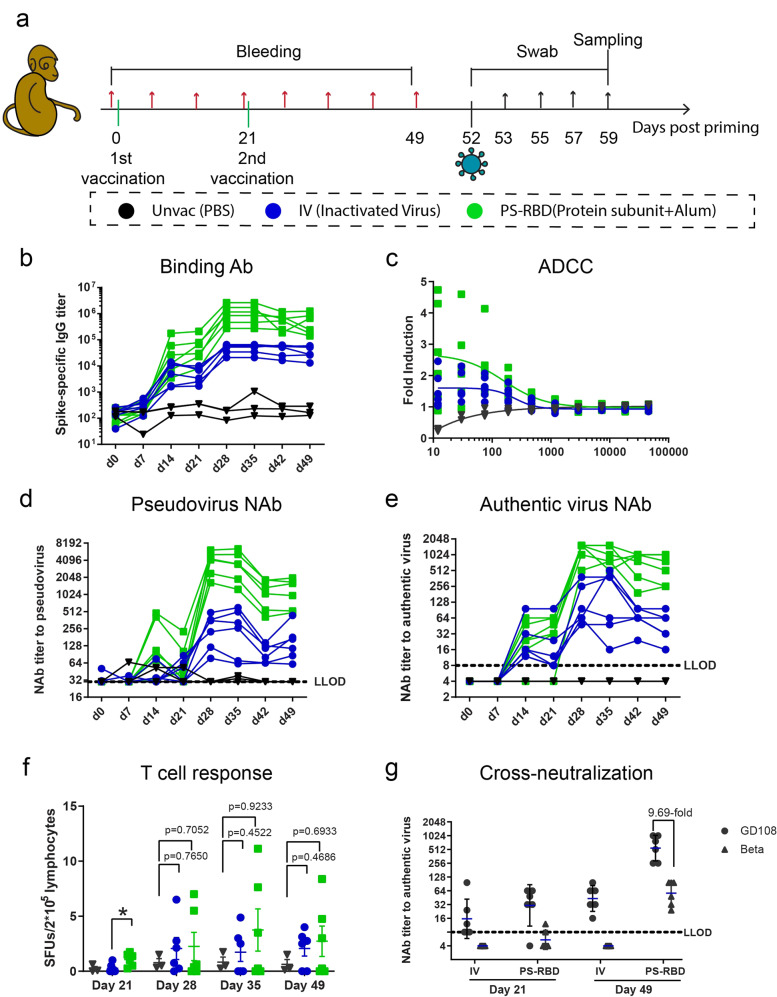
Immune responses induced by PS-RBD and positive control. **a** Schematic representation of the experimental protocol. Female rhesus macaques were randomly classified into 3 groups and immunized with 2 doses of PBS (Unvac, *n* = 3), inactivated virus vaccine (IV, *n* = 6), or RBD targeting protein subunit vaccine (PS-RBD, *n* = 6). “Unvac” was set as the blank control. For all NHPs, blood was collected before immunization and every 7 days post priming. On day 52, NHPs were challenged with Beta variant (B.1.351). Nasal swabs were collected at a dedicated time point as shown, and all NHPs were sacrificed on day 7 post challenge for the collection of the trachea, lung, and BALs**. b**–**e** Humoral immune responses, including the dynamic titer of sera spike protein-specific IgG (**b**), ADCC effect on day 42 (**c**), dynamic NAb titer measured by pseudovirus (**d**), and live prototype strain GD108 (**e**). Levels of ADCC were expressed as fold induction of relative light unit; NAb titers were expressed as 50% effective dilution (ED50) of serum. **f** Spike-specific T cell responses of PBMC were measured by IFN-γ ELISPOT assay and expressed as spot-forming units per 2 × 10^5^ lymphocytes. **g** Cross-neutralizing activity of sera to prototype (GD108) and Beta (B.1.351) is expressed as ED50. One spot represents one animal. Bars represent the mean ± SEM

To evaluate the immunogenicity of protein subunit vaccine in NHPs, groups of six female Chinese rhesus macaques, 2–3 years in age, were immunized with two doses of a dimeric RBD vaccine PS-RBD (25 g/dose) or an inactivated SARS-CoV-2 virus vaccine IV (5 g/dose) on days 0 and 21. Rhesus macaques intramuscularly injected with PBS (Unvac) were used as blank controls. Sera and peripheral blood mononuclear cells (PBMCs) were collected before and every 7 days after priming (Fig. 1a). All NHP sera converted positive after the first dose and reached a peak on days 14 and 21 in the IV and PS-RBD groups. The peak geometric mean titer (GMT) of anti-spike antibody after the first dose was 5373 in the IV group and 42,847 in the PS-RBD group. Anti-spike IgG was highly boosted after the second dose of IV and PS-RBD, and the GMT peaked on day 28, reaching 45,414 and 9,13,569, respectively (Fig. 1b). For neutralizing antibodies in sera, both pseudovirus and authentic virus neutralization assays were conducted. Similar to spike-specific IgG responses, the GMTs of serum NAb titers peaked around days 14–21 after priming and were highly boosted after the second dose to 226 (pseudovirus) and 124 (authentic virus) for IV, and 3613 (pseudovirus) and 1195 (authentic virus) for PS-RBD on day 28 (Fig. 1d, e). Both NAb titers to pseudovirus and authentic virus in the IV and PS-RBD groups decreased by almost half from day 21 to day 42; thereafter, the GMT levels to the authentic virus were maintained at 43 in the IV group and 548 in the PS-RBD group on day 49 (Fig. 1d, e). Both pseudovirus and authentic virus NAb titers were correlated with spike-specific IgG titers (*p* < 0.0001) with *r* = 0.9299 and *r* = 0.8702, respectively, and authentic virus NAb titers were correlated with pseudovirus NAb titers (*p* < 0.0001) with *r* = 0.8801 (Supplementary Fig. 1).

Furthermore, we analyzed the ADCC in the sera of NHPs. Results revealed that the ADCC activity calculated by subtraction of negative sera was significantly triggered in the IV and PS-RBD groups than in the Unvac control; the fold induction by 1:12 dilution was 1.53 for the IV group and 2.68 for the PS-RBD group (Fig. 1c).

Despite exogenous vaccine-like subunit vaccine and inactivated vaccine mainly inducing humoral immunity owing to the lack of MHC-1 pathways, a small number of peptides degraded from exogenous antigens could be cross presented by MHC-1 molecules to activate CD8 T cell responses.^29^ To investigate T cell responses, PBMCs were collected and stimulated by spike peptide pools in vitro for 20 h, after which an INF-γ ELISPOT assay was performed. In our results, the average quantity of INF-γ-secreting cells in the IV and PS-RBD groups was less than 5 spots per 2 × 10^5^ lymphocytes on days 21, 28, 35, and 49. The moderate level of SFUs in the two groups indicated that both vaccines administered in NHPs dominantly induced antibody responses rather than T cell responses (Fig. 1f).

To assess the neutralizing activity in vaccinated NHP sera against Beta variant, the neutralizing NAb titers against Beta variant (B.1.351) were measured by a microcytopathogenic effect assay on days 21 and 49 (Fig. 1g); Compared to the neutralizing activity against the prototype strain GD108, GMTs of NAb titers for Beta variant elicited by PS-RBD were still robust (GMT: 57, range: 24–96) on day 49, though 9.69-folds reduce compared with GD108. In the IV group, GMT of NAb titers on day 49 was also significantly decreased for the Beta variant with 6 out of 6 dropping lower than LLOD. NAb GMT induced by PS-RBD was significantly higher than that induced by IV against GD108 (*p* = 0.0022), and Beta (*p* = 0.0014) (Fig. 1g). This suggested that the current Beta variants escaped the immunity of NHPs induced by PS-RBD and IV in some degree.

### Viral load in nasal swabs, lungs, and bronchoalveolar lavages

**Fig. 2:**
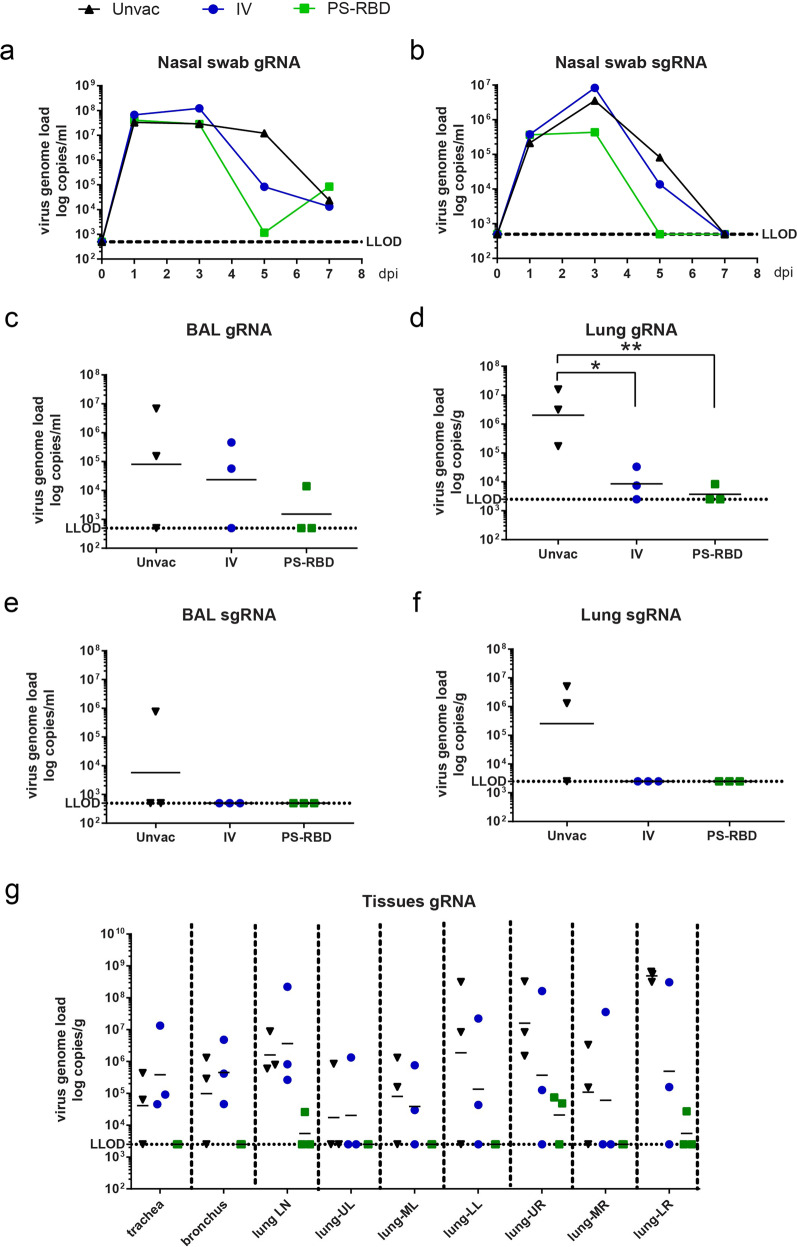
Viral load in rhesus macaques challenged with Beta variant B.1.351. Nasal swab, trachea, bronchus, lung LN, BALs, and lung lobe specimens were collected at the time shown in Fig. 1a. **a**–**g** SARS-CoV-2 gRNA and sgRNA levels were measured by RT-PCR and expressed as viral copies. LLOD refers to a low level of detection. gRNA was quantified in nasal swabs (**a**), BALs (**c**), a mixture of 6 lung lobes (**d**), trachea/bronchus/lung LN/each lung lobe (**g**). sgRNA was quantified in nasal swabs (**b**), BALs (**e**), and a mixture of six lung lobes (**f**). One spot represents one animal. *p < 0.05, **p < 0.01

Among the VOCs of SARS-CoV-2, the Beta variant was more difficult to neutralize by the anti-sera antibodies induced by vaccines targeting the prototype strain in our study (Fig. 2) as well as in other reports.^16–19^ To investigate whether multi-fold reduced NAb titers of NHP sera still confer sufficient protection against the Beta variant, 1 × 10^6^ TCID_50_ B.1.351 was applied to challenge NHPs by intranasal (IN) and intratracheal (IT) routes 4 weeks after the second dose of PS-RBD or IV (Fig. 1a). In this study, three out of six animals were challenged, and the rest macaques were retained for another study. Nasal swabs were collected from the macaques at indicated time points. On day 7 post-infection (dpi), lung tissues, bronchoalveolar lavage (BAL) fluid, trachea, and lung lymph nodes were collected from each macaque (Fig. 1a). SARS-CoV-2 genomic RNA (gRNA) and sub-genomic RNA (sgRNA) levels representing the replicating virus were measured by reverse transcription-polymerase chain reaction (RT-PCR) for each sample. Viral gRNA in nasal swabs of the three groups peaked at 1–3 dpi, whereas viral sgRNA peaked at 3 dpi and began to decrease thereafter (Fig. 2a, b). For overall quantification of viral RNA in nasal swabs, the area under the curve of log transferred viral RNA copies was calculated, and the median level of the PS-RBD group (gRNA:19.82; sgRNA: 10.18) was found to be lower than that of the IV group (gRNA: 24.33; sgRNA: 15.62) and Unvac control (gRNA:27.21; sgRNA: 16.08) (Fig. 2a, b). Specifically, reduced nasal viral copies were observed in the PS-RBD group on 5 dpi, with median reductions of 3.92 log10 gRNA copies/ml (2/3 lower than the lower limit of detection (LLOD)) and 2.33 log10 sgRNA copies/ml (3/3 lower than LLOD) compared to that in blank control (Supplementary Fig. 2). Relatively less reduction of viral load in nasal swabs on 5 dpi was found in the IV group, with a median reduction of 2.06 log10 for gRNA and 0.89 log10 for sgRNA. A similar trend was observed for lungs and BALs. PS-RBD vaccination reduced viral gRNA by 1.72 log10 in BALs (Figs. 2c) and 2.74 log10 in lungs (Fig. 2d). The Median gRNA load in BALs and lungs of the IV group was slightly lower than that in the blank control. Specifically, the viral load detected in all respiratory tissues in the PS-RBD group was lower than that in the Unvac control (Fig. 2g). For the IV group, the viral gRNA in five out of six lobes was lower than that of the Unvac control, but equivalent in the trachea, bronchus, and lung lymph nodes (Fig. 2g). In terms of lung and BAL sgRNA levels, both vaccinated groups showed values below LLOD, significantly lower than that in the blank control (Fig. 2e, f).

### Lung pathology and immunofluorescence assay

Seven days post Beta-variant challenge, macaques were euthanized and necropsied. Severe gross lesions were observed in the lung lobes of all animals in the unvaccinated group, including massive pulmonary punctate hemorrhage, swollen hilar, and mediastinal lymph nodes. For vaccinated groups, the aforementioned gross lesions were reduced in macroscopic view. Especially in the PS-RBD group, pulmonary punctate hemorrhage was not found in 3/3 macaques.

**Fig. 3:**
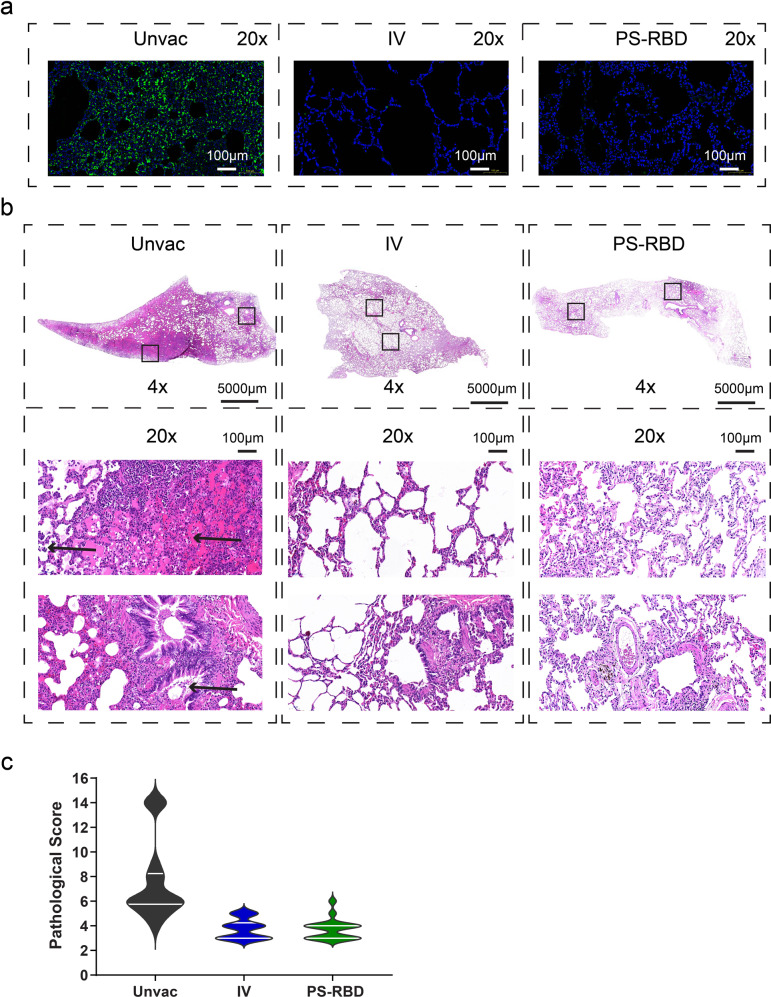
Histopathology and immunofluorescence of lungs on day 7 after challenge. **a** Representative graph of immunofluorescence of nucleocapsid protein (green) in lung tissue. **b** Representative graph of hematoxylin and eosin (H&E) staining of lung tissues. Arrowhead indicates the histopathological lesion. **c** Pathological score of all lung lobes of each macaque (18 specimens for each group) was counted according to the severity and extent of the histopathological lesion and represented as a violin plot

Lung sections were subsequently excised and subjected to histopathological and immunofluorescence assay for nucleocapsid protein. All animals in the unvaccinated groups developed multifocal interstitial pneumonia characterized by diffuse hemorrhage in the lung, thickening of the pulmonary septum, and infiltration of inflammatory cells. One out of three macaques in Unvac group developed severe interstitial pneumonia. Specifically, the alveolar spaces of this macaque were expanded (edema) and filled with cell homogenous, eosinophilic, proteinaceous fluid, admixed with necrosis fragment, fibrin, neutrophils, lymphocytes, enlarged alveolar macrophages, and detached type II pneumocytes. In distal bronchioles, degeneration and sloughing of epithelial cells were present (Fig. 3b). Compared to the unvaccinated groups, the pathological changes of vaccinated groups were much less severe (Fig. 3b), with an average pathological score of 3.72 for PS-RBD and 3.78 for IV, relative to 7.5 for the unvaccinated group (Fig. 3c). For immunofluorescence assay, substantial nucleocapsid protein of SARS-CoV-2 was detected in lung tissues of the unvaccinated control group, whereas scarce positive cells were detected for PS-RBD and IV groups (Fig. 3a).

These results demonstrated that the Beta-variant challenge could lead to typical interstitial pneumonia in rhesus macaques, meanwhile, both PS-RBD and IV could control the replication of the virus and provide protection against the variant-caused pathological injury.

### Immune activation in blood, lung, and bronchoalveolar lavage post infection

**Fig. 4:**
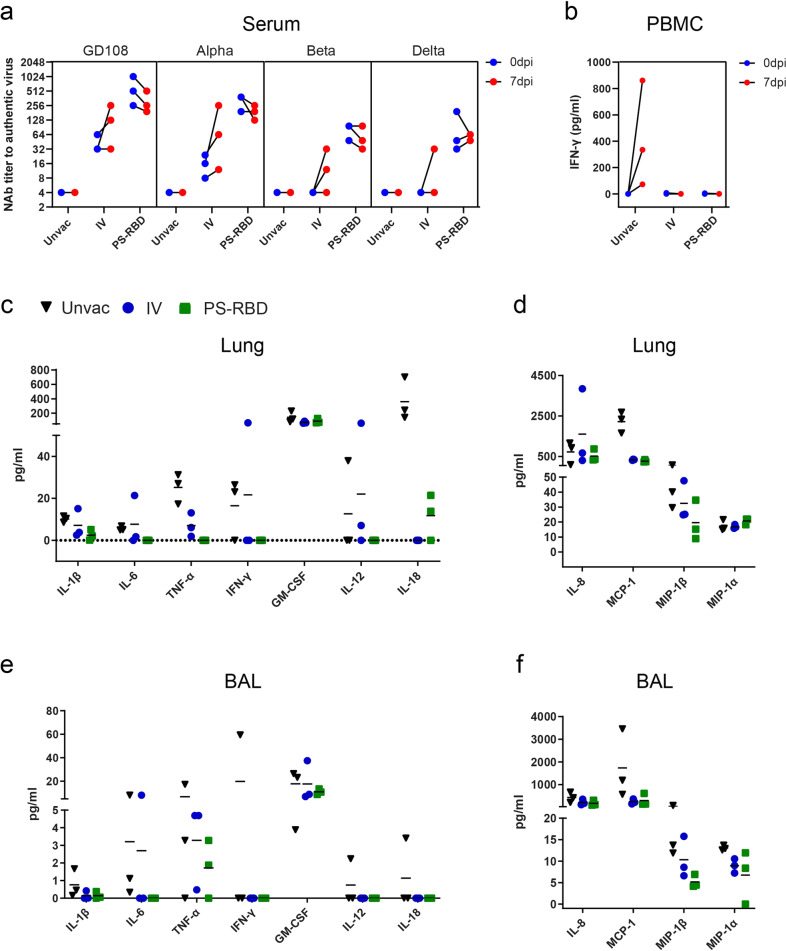
Immune status and Inflammatory cytokine analysis after challenge in different vaccine groups. **a** Serum neutralizing antibody titer against different VOCs before (0 dpi) and after (7 dpi) Beta variant challenge. **b** PBMCs were isolated before (0 dpi) and after (7 dpi) Beta variant challenge, and stimulated by spike peptide pools. The INF-γ concentrations of supernatant were quantified and presented. **c**–**f** Multiple inflammatory cytokines in BALs and lung tissue homogenate were measured and expressed as concentrations. The concentration of pro-inflammatory cytokines, such as IL-1β, IL-6, TNF-α, IFN-γ, IL-8, IL-12, and IL-18, in lung tissues (**c**) and BALs (**e**). The concentration of chemokines, including IL-8, MCP-1, MIP-1β, and MIP-1α, in lung tissues (**d**) and BALs (**f**). One spot represents one animal

Immune memory formation and recalled immune responses are important indicators for the effectiveness of administered vaccines. To characterize the immune responses recalled by infection in vaccinated NHPs, NAb, and T cell responses in blood were compared before and after the viral challenge. NAb titers on 0 and 7 dpi were tested against prototype strain), Alpha strain, Beta strain, and Delta strain. We found that the NAb titers to Alpha variant were elevated in all macaques in the IV group after the challenge, and those to prototype strain, Beta strain, and Delta strain were elevated in 2/3 macaques in the IV group (Fig. 4a). Specifically, the GMT level increased by 2.52-folds for prototype strain, 4.00-folds for Alpha strain, 2.88-folds for Beta strain, and 4.00-folds for Delta strain. Interestingly, for the PS-RBD group with higher NAb titers on 0 dpi, the NAb titers decreased by 0.57–0.89 folds on 7 dpi compared with 0 dpi for each macaque (Fig. 4a). Despite the antibody response, the spike-specific T cell responses before and after the challenge were further quantified in this study. PBMCs were isolated from blood samples and stimulated by a spike peptide pool, and the supernatants were collected and tested for IFN-γ. As a result, no IFN-γ was detected in the vaccinated and unvaccinated group on 0 dpi. On 7 dpi, IFN-γ in 3/3 macaques increased to 861 pg/ml, 334 pg/ml, and 72 pg/ml, whereas that was not induced in the IV group and PS-RBD group (Fig. 4b).

Inflammation is mediated by a variety of cytokines, such as interleukin-1 (IL-1), interleukin-6 (IL-6), and tumor necrosis factor-α (TNF-α). We assessed the main proinflammatory cytokines in the lungs and BALs. As per our results, PS-RBD generally downregulated the level of proinflammatory cytokines compared to that in the Unvac control, including IL-1β, IL-6, TNF-α, IFN-γ, interleukin-12 (IL-12), and interleukin-18 (IL-18) in the lungs and BALs (Fig. 4c, e). In the IV group, a significant reduction was only observed for TNF-α and IL-18 in BALs (Fig. 4e). Proinflammatory cytokines like monocyte chemotactic protein-1 (MCP-1, also known as CCL-2), macrophage inflammatory protein-1α (MIP-1α, also known as CCL-3), and macrophage inflammatory protein-1β (MIP-1β, also known as CCL4) are cytokines belonging to the CC chemokine family that is involved in the recruitment and activation of macrophages, while interleukin-12 (IL-8), also known as CXCL8, is involved in the recruitment of neutrophils. Interestingly, the average levels of MCP-1, MIP-1α, MIP-1β, and IL-8 in the BALs of PS-RBD and IV groups were lower than those in the Unvac group (Fig. 4f). For Lungs, MCP-1 and MIP-1β in the two vaccinated groups were also found to be significantly lower than that in the unvaccinated group (Fig. 4d).

The results demonstrated that the NAb titers could be recalled after infection in the IV group but not in the PS-RBD group with stronger pre-existing neutralization ability. Furthermore, the immune system was not overactivated after challenge in vaccinated groups as pro-inflammation cytokines were generally lower in vaccinated than the unvaccinated group.

### Cross-reactivation to all VOCs induced by PS-RBD

**Fig. 5:**
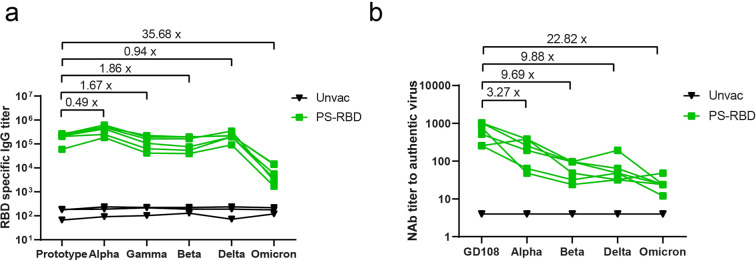
Cross-reactivity to all VOCs induced by PS-RBD. **a** Serum of the PS-RBD group were collected on day 49 and tested by ELISA for specific IgG against recombinant RBD of prototype, Alpha, Beta, Gamma, Delta, and Omicron variant. **b** Serum of PS-RBD group on day 49 was tested for cross-neutralizing antibody against GD108(prototype strain), Alpha, Beta, Delta, and Omicron strain

To estimate the potential protective effect of PS-RBD against various VOCs including the newly identified variant Omicron, sera of PS-RBD group on day 49 were tested for cross-binding and neutralizing activity to prototype, Alpha, Beta, Gamma, Delta, and Omicron strains. As a result, PS-RBD vaccinated sera could successfully recognize the recombinant RBD of all aforementioned VOCs (Fig. 5a). Specifically, RBD-specific IgG titers induced by PS-RBD changed slightly for Alpha, Beta, Gamma, and Delta strain compared with a prototype with a fold change of binding antibody GMT ranging from 0.49 to 1.86. Whereas, RBD-specific IgG titer induced by PS-RBD significantly decreased by 35.58-folds for Omicron strain compared with the prototype (Fig. 5a). similar to RBD-specific IgG titer, PS-RBD vaccinated sera could neutralize all various authentic VOCs, but reduce to varying degrees for different variants. Compared with NAb GMT against prototype, the NAb GMTs decreased by 3.27-folds, 9.68-folds, 9.88-folds, and 22.82-folds against Alpha, Beta, Delta, and Omicron strain, respectively (Fig. 5b). Therefore, macaque serum induced by PS-RBD still possessed effective binding and neutralizing activity against all current defined VOCs, which might demonstrate the potential protectiveness of PS-RBD against current VOCs.

## Discussion

Since variant strains of SARS-CoV-2 are prevalent worldwide and vaccine-induced NAb titers gradually decrease over time, it is urgent to evaluate the impact of mutant strains and NAb levels on vaccine protection at the earliest. In this study, one RBD-targeted vaccine and an inactivated vaccine were evaluated for cross-neutralizing reactivity and protective effect in a rhesus macaque model. Results showed that the NAb GMT of PS-RBD against the Beta and Delta variants was reduced approximately tenfold compared with that against GD108, a prototype strain isolated early in 2020; however, it still conferred significant protection upon Beta-variant challenge, characterized by a reduction in lung viral load, pathological change, and inflammatory response. The inactivated virus vaccine used in this study elicited lower NAb titers against Beta variants that were below LLOD, but still provided slight protection against the Beta-variant challenge, indicating the possibility of other correlates of protection being present. Furthermore, cross-reactivity to Alpha, Beta, Gamma, Delta, and Omicron was induced by PS-RBD in macaques, indicating potential effective protection of PS-RBD for all VOCs.

Studies have shown that NAb against different VOCs induced by the vaccines developed in an emergency is reduced by varying degrees.^16–23,30,31^ PS-RBD evaluated in this study is a protein subunit vaccine using a dimeric form of the receptor-binding domain (RBD) as the antigen, the antibodies generated by the hosts should definitely recognize the different VOCs sharing a common sequence. However, the NAb levels induced by PS-RBD were highly divergent for different VOCs due to different mutation sites existing in each variant. For the most immune escaped variant Omicron, 15 mutation sites were identified, and preliminary structural analysis indicated that amino acid residue mutations at positions 477, 493, 496, 498, 501, and 505 on RBD affected the interaction with ACE2.^32^ E484 site is a key immune escape site that has been clearly studied,^33,34^ and mutation at E484 site is comprised in several VOCs, including Beta, Gamma, Delta, and newly defined VOC Omicron. In our study, antibody-induced by PS-RBD was still able to recognize Alpha, Beta, Delta, and Omicron strain, though nearly 3- to 22-folds reduction for NAb titer was measured compared with that against prototype strain, suggesting a reduced but still potential protection against all current VOCs provided by PS-RBD.

As described in the result section, NAb titers against Beta and Delta variants induced by PS-RBD significantly decreased compared to that against GD108 and Alpha variants. In order to demonstrate whether PS-RBD could still effectively protect against variants with higher escaped ability, we chose the Beta variant to challenge animals and established the Beta-variant challenge model in rhesus macaques for the first time. Similar to the results published by Lu et al.,^35^ Shan et al.,^36^ and Salguero et al.^37^ on the prototype strain challenge model, lung tissues of the Beta strain infected macaques showed typical gross lesions and pathological changes in interstitial pneumonia on 7 dpi, which was the set necropsy time. Viral replication in the upper respiratory tract peaked during 1–3 dpi, and gradually declined thereafter (Fig. 2a, b). The difference was that the sgRNA level of the Beta variant in the upper respiratory tract dropped below the LLOD on 7 dpi, which was lower than the data reported for the prototype strain; the replicating viruses were mainly concentrated in the lower respiratory tract. This might suggest a certain difference between the replication characteristics of prototype strain and that of the Beta strain in macaques.

Vaccine protection against Beta variant was estimated for protein subunit vaccine targeting RBD and inactivated vaccine in the study. Although NAbs against Beta variant induced by PS-RBD was reduced by approximately 10-folds, it reduced the viral gRNA copies of Beta strain by 3.92 log10 and completely eliminated the viral sgRNA in lung lobes, which met the evaluation criteria for preclinical effectiveness set by Center for Drug Evaluation of National Medical Products Administration.^38^ Overall, the ranked order of protective effect was unvaccinated < IV < PS-RBD according to viral load, pathology, and inflammatory factors, respectively. These data fully illustrated the effectiveness of PS-RBD protection against Beta variants in the NHP model. In addition, our data showed that PS-RBD significantly relieved the pathological changes and reduced the inflammatory cytokine levels in the respiratory tract and lung. Some studies showed that elevated levels of IL-6, TNF-α, MCP‐1, MIP-1α, and G‐CSF were correlated with severe COVID-19.^39–41^ Pro-inflammation cytokines such as IL-1β, IL-6, and TNF-α, bind to their receptors, further mediate inflammation through triggering intracellular signaling pathways, including the nuclear factor kappa-B (NF-κB), mitogen-activated protein kinase, and Janus kinase-signal transducer and activator of transcription pathways. To illustrate whether the excessive immune response was induced by PS-RBD and inactivated vaccine, multiple pro-inflammatory cytokines in lung and BALs were measured and analyzed. In our results, general pro-inflammation cytokines, including IL-1β, IL-6, TNF-α, IL-8, IFN‐γ, were relatively lower than the unvaccinated control group. General level of those cytokines in the IV group was shown to be in the middle of the two. IL-8, MCP‐1, MIP-1α, and MIP-1β were chemokines recruiting mononuclear macrophages and neutrophils, which are the main cells mediating acute inflammation. In our study, these pro-inflammation chemokines in lung and BAL of macaques were generally downregulated in PS-RBD and IV group, among them, relatively lower in the PS-RBD group. It might be one of the reasons for better prognosis of vaccinated macaques. Furthermore, for unvaccinated macaques, the level of IFN-γ secreted by spike specific T cells in PBMC highly increased after infection, indicating a pathogenic T cell response was triggered by SASR-CoV-2 to secrete pro-inflammation cytokines. Whereas, such elevation was not found in vaccine groups, indicating vaccination might avoid the over-activation of pathogenic immune responses after SARS-CoV-2 infection.

Neutralizing ability of PS-RBD against the Beta strain and prototype strain was higher than that of the inactivated virus vaccine while the lung viral load was significantly lower than that of the latter, suggesting neutralizing antibodies might be the main CoP against SARS-CoV-2.^42^ As per our results, the binding antibody, pseudo-virus NAb, and authentic virus NAb showed good linear correlations with each other (Fig. S1). Thus, it would be feasible to define the GMT of binding antibody, pseudo-virus, or authentic virus NAb as a surrogate end-point or protection threshold. Interestingly, although the NAb level of inactivated virus vaccine group decreased to an extremely low level for the Beta variant (GMT < 8), it still exerted a certain protective effect against the Beta variant with a reduction of the viral load by 0.25–2.99 log10 in 5/6 lung lobes, although no reduction was seen for lung LN, trachea, and bronchus. In this study, neither PS-RBD nor inactivated virus induced a moderate T cell response before and after challenge, with only several spot forming units per 2 × 10^5^ cells after subtracting the unstimulated background. It indicated that there might be other correlates involved in inactivated vaccine induced protection. ADCC is one of the antibody-regulated immune responses mediated via the Fc fragment that might contribute to virus elimination.^43–46^ It has been investigated in patients with COVID-19, and was found to be higher in the ones recovered from the disease than in those who deceased.^47^ In our study, the ADCC effect triggered by inactivated virus vaccine in the NHP model was significantly higher than that in the unvaccinated control, but lower than that in the PS-RBD group. Furthermore, immune memory and the recalled NAb responses were also evaluated in our study. As a result, NAb titers in inactivated group were highly boosted after Beta variant challenge, whereas, that in PS-RBD group remained slowly declining. We supposed that PS-RBD group with high level of pre-existing NAb might inhibit the viral replication at initial stage of infection, thus scarce spike protein was expressed by lung cells to trigger antibody response. For inactivated vaccine, though with lower level of NAb, the immune system could quickly respond to viral infection and immediately elevate the antibody level to control viral infection as the immune memory status developed. Those results suggested that the ADCC and immune memory might be other correlates of COVID-19 vaccine induced protection; however, further research would be required in this regard.

In summary, the current study established a rhesus macaque model of infection using the Beta variant (B.1.351) to evaluate the immune protective effect of the RBD targeted protein subunit vaccine and inactivated whole virus particle vaccine. Both evaluated vaccines in this study significantly reduced the viral load in respiratory tract, alleviated pathological changes, and relieved lung inflammation in vaccinated macaques. The protective effect of vaccines was in the order PS-RBD > inactivated vaccine > unvaccinated, according to the aforementioned indices. We specified the cross-reactivity to all current VOCs induced by the RBD-targeting protein subunit vaccine used in the study and demonstrated potential protective ability of this vaccine against all these VOCs forementioned. Furthermore, this study highlighted the possible contribution of antibodies possessing neutralizing reactivity or cell-mediated cytotoxicity, and immune memory to vaccine induced protection.

## Materials and methods

### Animals and ethics statement

Chinese rhesus macaques (2–3 years old, female) were provided and housed in the Kunming National High-level Biosafety Primate Research Center, China. All experiments with live SARS-CoV-2 in the NHP model were performed in the ABSL-4 facility. All animal studies were conducted under the relevant ethical regulations and were approved by the Institutional Animal Care and Use Committee of Institute of Medical Biology, Chinese Academy of Medicine Sciences & Peking Union Medical College, China (Approval Number: DWSP202103004).

### Vaccination and challenge experiment

A total of 15 young Chinese rhesus macaques of good health were intramuscularly immunized with two doses of either 25 μg PS-RBD (*n* = 6), 5 μg (*n* = 6) inactivated vaccine, or PBS (*n* = 3) by 14 days interval. PS-RBD is a protein subunit vaccine using a dimeric form of the RBD as the antigen, and the inactivated vaccine used in this study was prototype virus strain that isolated from Wuhan patient in early 2020 and inactivated by formalin. Both vaccines used in the study were adjuvanted by Aluminum. Blood samples were collected before immunization and every 7 days post priming; antibody responses were measured thereafter. PBMCs were isolated for T cell response detection at day 21, 28, and 49. On day 52 after the initial vaccination (4 weeks after the 2nd dose), all animals were challenged with Beta variant (B.1.351) of 1 × 10^6^ TCID_50_ via intratracheal and intranasal routes (1:1). The specimens were collected as shown in Fig. 1a.

### ELISA for estimating total spike-specific IgG

The levels of serum antibodies directed against SARS-CoV-2 spike protein were assessed using an ELISA-based assay. Totally, 96-well EIA/RIA plates were coated overnight with 1 µg/mL recombinant spike protein at 4 °C. After removal of unbound spike protein and blocking the plate with 10% fetal bovine serum in 0.5% PBST, 10-fold serially diluted test samples were added to the wells. Bound antibodies were subsequently detected after incubation with 1:5000 diluted goat anti-mouse IgG (HRP-labeled) (China ZSGB-BIO, cat#ZB2305), followed by development with the substrate (China Beijing Wantai BioPharm, cat#N20200722) at 450 nm and 630 nm. The endpoint of serum antibody titers was determined by reciprocal of the highest dilution, which was 2.1-fold higher than the optical absorbance value of the negative control.

### Serum neutralization assay

Levels of serum NAbs against SARS-CoV-2 were measured using live and pseudo-SARS-CoV-2 viruses, and the results were expressed as GMT. NAbs against live SARS-CoV-2 were quantified using a microcytopathogenic effect assay at a minimum of eightfold dilution. The neutralization capacity against pseudovirus (Wuhan-Hu-1, GenBank: MN908947, optimized for human cell expression) was determined following a previously reported protocol.^48^

### IFN-γ enzyme-linked immunospot assay

Freshly isolated splenocytes were stimulated with a peptide pool spanning the SARS-CoV-2 spike protein for 20 h at 2 × 10^5^ cells per well. The concentration of each peptide was 5 μg/mL. The peptide pool was generated as follows: a panel of consecutive 15-mer peptides with overlapping 9 amino acids were synthesized to encompass the entire spike protein and mixed as one peptide pool. After stimulation, the plates were incubated with IFN-γ-detecting antibodies. Spots representing IFN-γ-producing cells were enumerated using an ImmunoSpot S6 Universal Reader, CTL. Final determinations were calculated by subtracting the negative stimulation background levels from the measured values.

### Antibody-dependent cell-mediated cytotoxicity

To evaluate antibody-dependent cell-mediated cytotoxicity (ADCC), Jurkat-hFcγRIII-NFAT-Luc reporter cells were used as ADCC bioassay effector cells, and 293FT cells with stable expression of SARS-CoV-2 spike protein were used as ADCC bioassay target cells.^49^ Both cell lines were obtained from the Chinese National Institutes for Food and Drug Control.

For all ADCC assays, target cells (4.25 × 10^3^ per well) were incubated with effector cells (7.5 × 10^4^ per well) and serial dilutions of serum samples were performed at 37 °C for 16 h. After incubation, the relative light unit (RLU) was detected according to the instructions provided by PerkinElmer (Waltham, MA). Fold of induction (FI) was calculated as follows: FI = RLU (induced − background)/RLU (negative serum control − background).

### Quantification of SARS-CoV-2 genomic RNA and sub-genomic RNA

At the dedicated time shown in Fig. 1a, specimens of the nasal swab, trachea, bronchus, lung lymph nodes, BALs, and 6 lung lobes were collected for virus RNA detection. Tissues were weighed, homogenized, and clarified by centrifugation at 8000 rpm for 10 min at 4 °C, and supernatants were obtained therefrom. Viral RNA was extracted using a magnetic viral nucleic acid kit (TIANGEN, China) according to the manufacturer’s protocol. Viral genomic RNA (gRNA) in each sample was quantified by RT-PCR targeting the ORF1ab and N genes of SARS-CoV-2. For sub-genomic RNA (sgRNA) quantification, RT-PCR was performed targeting the sequence overlapping ORF1ab and E genes of SARS-CoV-2. The viral load in the sample was quantified according to a standard curve. The LLOD of viral RNA was set as 1 copy/μl because the log copies could not be accurately measured when under 1 copy/μl by the assay. The LLOD was transformed to 2500 copies/g for lung and 500 copies/ml for BAL and swabs according to the volume of RNA extraction reagent.

### Histopathology

Lung pathology was evaluated by hematoxylin and eosin (H&E) staining, as previously described.^35^ Five-micron-thick formalin-fixed paraffin-embedded sections were prepared and stained. The slides were evaluated by a pathologist in a double-blind manner. The pathological scores were calculated by a pathological scoring system established by the Institute of Medical Biology, Chinese Academy of Medicine Sciences. The scoring system was developed according to the typical pathological changes of SARS-CoV-2 reported in Lu et al’s study^35^ and mainly referred to the scoring template of SARS-CoV described in Liu et al’ work.^50^

### Multiple cytokine profiling

Lung lobe and BALs were collected on day 7 post challenge. Lung-lobe specimens were weighed, and supernatants of the homogenate were prepared for detection. Pro-inflammatory cytokines, including IL-1β, IL-6, TNF-α, IFN-γ, IL-8, IL-12, IL-18, MCP-1, MIP-1β, and MIP-1β in all supernatants and BALs were measured by MILLIPLEX MAP NHP Cytokine Magnetic Bead Panel—Immunology Multiplex Assay. The concentration of each sample was calculated using a standard curve.

### Statistical analysis

Antibody titers were transformed into log10 values to calculate geometric means. All statistical analyses were conducted using GraphPad Prism v.7.0 (GraphPad Software, Inc.). Comparisons between two groups were tested using a two-tailed *t* test, while that across multiple groups was performed using one-way analysis of variance. Statistical significance was defined as *p* < 0.05.

## Supplementary information

Supplementary Materials

## Data Availability

All data generated in this study are available from the corresponding author upon reasonable request.
