# Supplementary material for: Immunogenicity and protective efficacy of a recombinant protein subunit vaccine and an inactivated vaccine against SARS-CoV-2 variants in non-human primates
Source: Signal Transduct Target Ther. 2022 Mar 3;7:69. doi: 10.1038/s41392-022-00926-y (PMC8892123; doi:10.1038/s41392-022-00926-y)
Supplement: Supplementary file 1 — Supplementary Materials [file 41392_2022_926_MOESM1_ESM.docx]

Supplementary Materials for

**Immunogenicity and protective efficacy of a recombinant protein subunit vaccine and an inactivated vaccine against SARS-CoV-2 variants in non-human primates**

Qian He†, Qunying Mao†, Xiaozhong Peng†, Zhanlong He†, Shuaiyao Lu†, Jialu Zhang, Fan Gao, Lianlian Bian, Chaoqiang An, Wenhai Yu, Fengmei Yang, Yanan Zhou, Yun Yang, Yanyan Li, Yadi Yuan, Xujia Yan, Jinghuan Yang, Xing Wu, Weijin Huang, Changgui Li, Junzhi Wang*, Zhenglun Liang*, Miao Xu*

Correspondence to: [xumiaobj@126.com; lzhenglun@126.com; wangjz@nifdc.org.cn;](mailto:xumiaobj@126.com;%20lzhenglun@126.com;%20wangjz@nifdc.org.cn;%20pengxiaozhong@pumc.edu.cn)

**This PDF file includes:**

Figures. S1 to S2

Figure. S1.


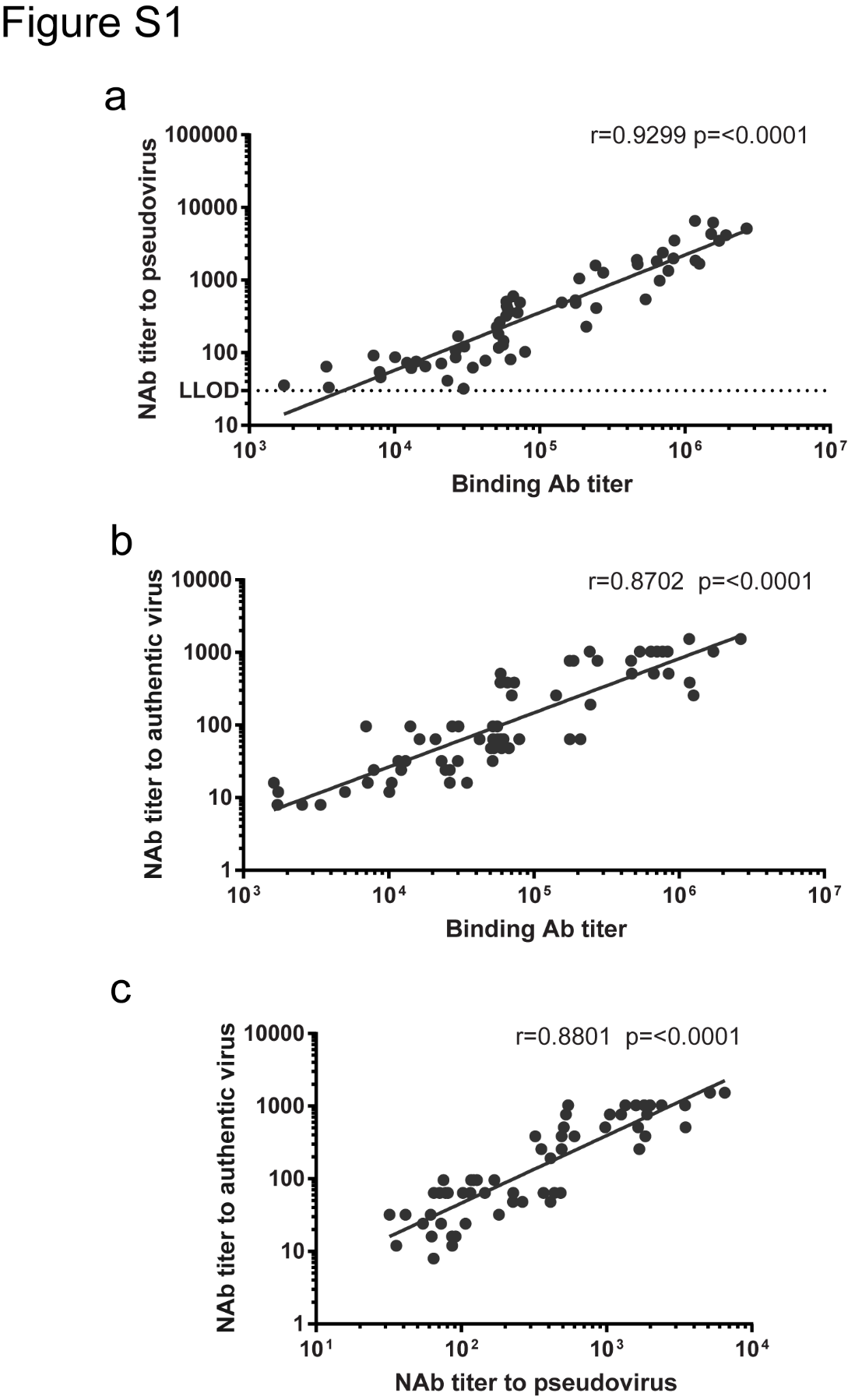


**Supplementary Figure 1. Correlation of spike-specific binding antibody titers and neutralizing antibody titers.** The spike-specific IgG level, pseudovirus NAb, and authentic virus NAb shown in Figure 1 were compared with each other. Pearson correlation coefficient was computed for the correlation between each other.

Figure. S2.


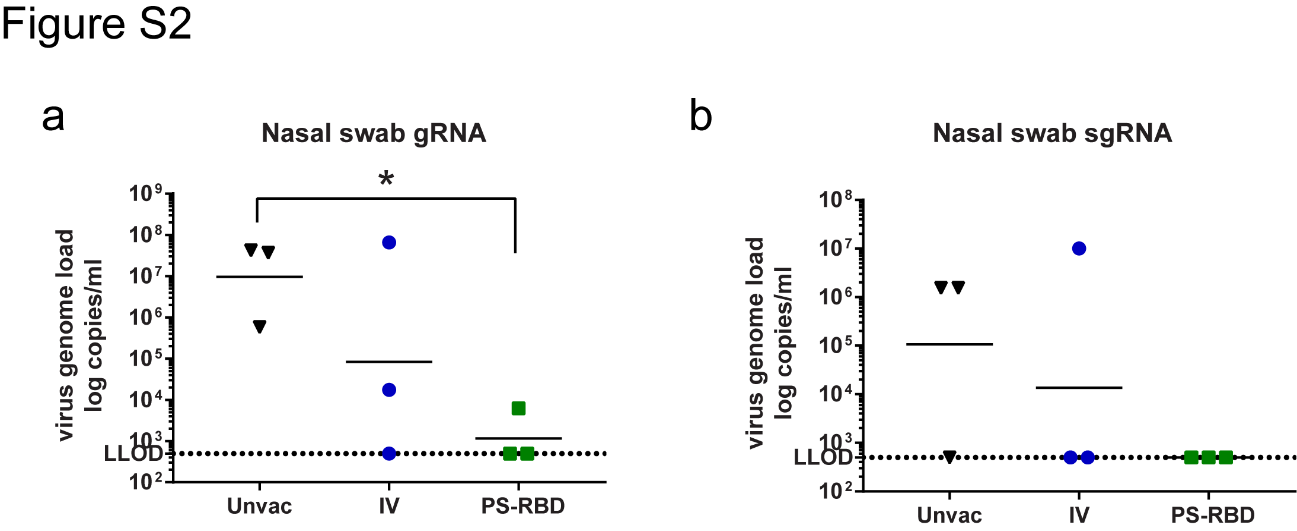


**Supplementary Figure 2. Viral load in nasal swabs collected on 5 dpi.** Nasal swabs were collected at the time shown in Figure 1a. gRNA and sgRNA levels in nasal swabs on day 5 post challenge were compared for each group.
